# Farther Observations on the Treatment of Hydrocephalus Internus

**Published:** 1799-10

**Authors:** Charles Brown

**Affiliations:** Surgeon


### *Farther Obſervations on the treatment of Hydrocephalus internus:—By* Mr. Charles Brown, *Surgeon.*

### *To the Editors of the Medical and Phyſical Journal.*

Gentlemen,

By your permiſſion, I continue my obſervations on the treatment of dropſy of the brain. Conſidering the treatment of *hydrocephalus internus* by no means ſo difficult as practitioners in general imagine, I preſume the following remarks will not be found unworthy of inſertion in your very uſeful publication, to which I wiſh all poſſible ſucceſs; and remain, with the greateſt reſpect,

Your moſt obedient ſervant,

No. 25, Hatton Garden, CHARLES BROWN.
*Sept. 7th,* 1799.

In reflecting, at various times, on the nature and treatment of hydrocephalus internus, ſome ideas have occurred to me which I have neither met with in converſation or books. Our imperfect knowledge of the ſtructure of the brain, and of the diverſified energy of the nerves, in their origin, progreſs, and termination, neceſſarily involves the diſorders of the head in a peculiar peculiar degree of uncertainty; and it is often extremely difficult to difcriminate even between the fympathetic and idiopathic affections of that important organ. It cannot, therefore, be furprifing, that the caufes of hydrocephalus have not hitherto been afcertained with any degree of accuracy or precifion. The light which diffections afford is obtained only at the clofe of the malady; and the ftate of the encephalon may have undergone confiderable changes, either by the operation of nature, or by the action of the medicines employed.

The *fymptoms* of this complaint are:

At the beginning a pain in the head, generally confined to one fide, efpecially above the eyes, and in a direction between the temples; then follow heavinefs, lofs of appetite, deafnefs, ficknefs at ftomach, coftivenefs, ftupor, and coma; there is fever, with a frequent, weak pulfe; the fkin is dry and hot, and there are frequent flufhings in the cheeks. In the commencement of the difeafe, the pupils are very much contracted; but as the difeafe advances, a dilatation of the pupil takes place, chiefly in that eye on which fide the fluid is collected. The child at intervals will fcream out, and have frightful dreams; latterly it will pick the bed-clothes, have *fubfultus tendinum*, and talk incoherently. In this ftate I lately attended a child (with a phyfician), who lingered out a fortnight, occafioning the moft poignant diftrefs to its parents. M. PETITE, in the "*Memoirs of the Academy of Sciences at Paris*," has remarked other fymptoms at the commencement of the difeafe, which are worthy of attention: thefe are—convulfive motions of the lips and eye-lids; biting the lips; picking the nofe; grinding the teeth; coftivenefs, or purging; languor of the eyes; palenefs; debility; heavinefs, and depreffion of fpirits; fleepinefs, with perpetual moaning; and fometimes inability to fupport the head upright. He obferves, that the difeafe comes on after *worms*, painful dentition, and violent convulfions. Dr. FOTHERGILL adds, fhort and difturbed fleep, and towards the clofe of the difeafe, urine and ftools come infenfibly away; the iris immoveable; the heat great; breathing fufpicious; the pulfe trembling, and quick beyond the poffibility of counting; after which a fpafm puts a period to the gloomy fcene.

The patients of M. Petite died convulfed, and he found water in the brain.

Hydrocephalus is diftinguifhed from apoplexy by its being attended with fever, and from nervous fever by the paroxyfms being very irregular, with perfect intermiffions, many times a day. In nervous fever, the pain in the head generally affects the middle of the head—in hydrocephalus it is ufually on one fide; and I agree in opinion with Dr. DARWIN, that the great difpofition poſition in perſons labouring under the diſeaſe to lye down immediately after having raiſed their heads from off the pillow, is owing to the preſſure of the water on the large trunks of the blood-veſſels entering the cavity, being more intolerable than on the ſmaller ones; for, if the large trunks are compreſſed, it muſt inconvenience the branches alſo; but if ſome of the ſmall branches are compreſſed only, the trunks are not ſo immediately incommoded: and I think it is highly probable, that where one eye is affected, the diſeaſe exiſts in the ventricle of that ſide. In the chief number of caſes of hydrocephalus I have had an opportunity of examining after death, I have found the fluid lodged in the cavities or ventricles of the brain. Authors have ſet it down as a great peculiarity, that the water has been collected within the brain in particular capſules. Once or twice it has been found above or upon the brain, between that and the finer *tunica* next to it; likewiſe between that *tunica* and the firmer exterior one, which alſo is ſaid to have been ſeparated from the interior vault of the cranium, and conſequently to have given room to water; but in theſe very rare caſes, the water has beſides been found in the ventricles of the brain, where it probably firſt has been collected, and from thence found an iſſue. The late Mr. John Hunter ſuppoſed, that the fluid was always collected in the cerebrum *only*, and that the cerebellum never had any ſhare in it. Children are ſometimes born with the bregma ſtretched, and a pulſation felt through it; and where this part remains long unoſſified, Aquapendente adviſes us to diſcharge the water at this place. But this is dangerous; for ſo ſuddenly taking off a preſſure the brain has long been accuſtomed to, may very likely kill the child. Hieronymus Mercurialis,* who wrote in the beginning of the ſixteenth century, was perhaps the firſt who mentions the diſeaſe as having its ſeat in the ventricles. Wepfer alſo juſt ſays that water has been formed in the cavities of the brain.† Boerhaave, Petite, and others have likewiſe ſpoken of it; but no author, I believe, deſcribed it at all accurately before Dr. Whytt, who expreſsly wrote on the internal watery head, anno 1768. But it has not been generally noticed, that the water lies ſometimes between the pia-mater, and the brain, as it is found to do in maniacs‡; and Dr. Underwood has met with it both there and in the ventricles, in the ſame ſubject, and always in infants under two years old.¶

*Of the Cauſes of Hydrocephalus.*

On this head there are various and oppoſite opinions. Dr. Percival ſuppoſes this complaint to ariſe moſt commonly from glandular obſtruction, and

* 'Opuſcula aurea, lib. de Morbis Puerorum.' † 'Histor. Apoplect.'

‡ 'Haslam, on Insanity.' ¶ 'Diseases of Children, vol. i. p. 272.'

and either general or local plenitude. Dr. QUIN imagined it to arise from preſſure on the brain, and fullneſs of the vaſcular ſyſtem from other cauſes. The remote cauſe is attributed by an able ſurgeon in this city (Mr. EDWARD FORD) to an inflammation of the veſſels of the pia-mater, which may owe its origin to the meaſles, ſmall-pox, ſcrofula, and other complaints, which may affect the brain in the ſame manner they do the meſenteric and other glands. As to any hereditary diſpoſition, no ſound reaſoning can be advanced in its favour*. I conſider dropſy in the head to ariſe in the ſame manner as in other parts of the body. The ventricles of the brain, as well as all other cavities of the ſolid parts, either larger or ſmaller, are kept ſmooth by a ſubtle, aqueous vapour, which continually perſpires from the blood-veſſels. It is eaſy to conceive, that though the vapour be ever ſo ſubtle and imperceptible, within a ſhort time, nevertheleſs, it would, not being carried off for ſome days, or months, be collected in a quantity, ſo as at laſt to diſorder and flow over the place. Providence has prevented the diſorder which would ariſe from hence, by furniſhing the brain with innumerable lymphatic veſſels, which being rouſed into action by proper and well-timed remedies, gradually abſorb the fluid, after which theſe veſſels carry it into the blood again, the ſuperfluous parts of it being depoſited and carried off by certain means provided for that purpoſe.

Under a ſtate of diſeaſe of the nature of dropſy, the vapour is more copiouſly effuſed, and a collection of water is perceptible; this is univerſally the caſe in dropſy in what part ſoever it has its ſeat; therefore the *cauſa proxima* may be a fault either in the tubes that carry the vapour to and from the place, or in the quality of the vapour itſelf. Accumulations of water ariſe in any part of the ſyſtem, whenever the internal veſſels of exhalation are relaxed and dilated, ſo that they perſpire more than a due quantity, and the proceſs of abſorption is more tardy. We know that moſt of the fluids ſecreted from the circulating maſs, and poured into cavities, may be abſorbed from theſe, and returned again by the lymphatics to the courſe of the circulation. But the ſame ſecreted fluids ſeem often to be returned alſo into the courſe of the circulation, or retrograde motion in the excretory and ſecretory veſſels †.—As the diſeaſe may originate from ſuch different cauſes, there can be no doubt but it may ſometimes be a chronic diſeaſe, and its appearances very inſidious.

[To be continued in our next Number.]

* I have already contended againſt hereditary diſeaſes, in my work on Scrofulous Affections; 8vo.

† Cullen's Phyſiology.

